# Effects of engineered aluminum and nickel oxide nanoparticles on the growth and antioxidant defense systems of *Nigella arvensis* L.

**DOI:** 10.1038/s41598-020-60841-6

**Published:** 2020-03-02

**Authors:** Azam Chahardoli, Naser Karimi, Xingmao Ma, Farshad Qalekhani

**Affiliations:** 1 Department of Biology, Faculty of Science, Razi University, Kermanshah, Iran; 2 Medical Biology Research Center, Kermanshah University of Medical Sciences, Kermanshah, Iran; 3 Zachry Department of Civil and Environmental Engineering, Texas A&M University, TAMU 3136, College Station, TX 77843-3136 USA

**Keywords:** Chemical biology, Plant sciences

## Abstract

The effects of different concentrations (0, 50,100, 1000 and 2500 mg/L) of engineered aluminum and nickel oxide nanoparticles (Al_2_O_3_ and NiO NPs) on plant growth, oxidative stress and antioxidant activities in the hydroponically grown tissues of *Nigella arvensis* L. were investigated. The plant biomass was significantly increased under 50 and 100 mg/L of Al_2_O_3_ NPs or 50 mg/L of NiO NPs treatment, but was significantly decreased at higher concentrations of these nanoparticles. Assays of several enzymatic antioxidants such as ascorbate peroxidase (APX), catalase (CAT), superoxide dismutase (SOD) and peroxidase (POD) in roots and shoots indicate a general increase of activities after exposure to 50–2,500 mg/L of Al_2_O_3_ NPs and NiO NPs. The results are corroborated by an increased 2,2-diphenyl-1-picryl hydrazyl (DPPH) scavenging activity, total antioxidant capacity, total reducing power, total iridoids content, total saponin content, and total phenolic content in treated plants by Al_2_O_3_ NPs compared to the control plants. By contrast, the antioxidant activities, formation of secondary metabolites, and other related physiological parameters such as the total antioxidant capacity, DPPH scavenging activity and total saponin content were inhibited after the concentration of NiO NPs was increased to 100 mg/L. Total phenols, saponins, iridoids and total antioxidant content and DPPH scavenging activity were increased in plants treated with 100–2,500 mg/L Al_2_O_3_ NPs. Overall, these two nanoparticles displayed different effects in the shoots and roots of plants at different concentrations, which may be due to their physico-chemical properties.

## Introduction

Rapid development of nanotechnology has greatly expanded the applications of engineered nanoparticles (ENPs) in commercial and industrial products^1^. Increased application and potential accumulation of ENPs in the environment and their unknown interactions with different organisms, aggravated by some reports of greater toxicity at nanoscale than the bulk scale, caused broad concerns about the environmental health and safety of ENPs^2,3^. Previous studies have demonstrated some disruptive effects of some ENPs on the natural environment including water, air, and soil quality^4^. Plants are one of the most essential components of the ecosystem and interact with ENPs closely^5^. These ENPs could be taken up by plants, and enter into the food chain through dietary consumption, ultimately affecting human health^6^.

Many previous investigations explored the potential applications of ENPs in agriculture^7–10^. However, the majority of previous studies in ENPs - plant interactions focused on the potential toxicity of nanoparticles to higher plants. Both positive and negative or insignificant effects of ENPs on plants have been reported^11^. In general, the phytotoxicity of ENPs is mediated through the production of reactive oxygen species (ROS) in plant cells^12^. Even though ROS are normally produced within plants as a byproduct of metabolic processes in chloroplasts and other organelles^13,14^, excessive production of ROS can disrupt plant photosynthesis and other physiological and biochemical processes, eventually triggering the defense mechanisms in plants such as greater activities of certain antioxidants^15^. In addition, accumulation of ENPs can stimulate defense mechanisms through antioxidant enzymes to scavenge the deleterious ROS^16^.

The amount of ROS and antioxidant responses vary with metal oxide nanoparticle types, exposure conditions and plant species^15^. For example, La_2_O_3_ NPs at 2,000 mg/L of induced significant ROS production and cell death in cucumber (*Cucumis sativus* L.)^17^. However, no significant effects was observed when cucumber was exposed to 0.2–200 mg/L CeO_2_ NPs^17^. Cerium oxide nanoparticles (CeO_2_NPs) increased H_2_O_2_ content in *Zea mays* and *Brassica rapa*^18,19^ but decreased the H_2_O_2_ content in *Oryza sativa*^20,21^. Hu *et al*.^22^ showed that SOD and CAT activities significantly increased in *Salvinia natans* treated with 50 mg/L ZnO NPs^22^. Kim *et al*.^23^ also reported high activities of SOD, POD and CAT following ZnO and CuO NPs (100 mg/L) applications to cucumber plants^23^.

Among the most abundant nanoparticles produced in the industrial and commercial sectors are aluminum oxide nanoparticles (Al_2_O_3_ NPs) and nickel oxide nanoparticles (NiO NPs). Al_2_O_3_ NPs are widely used in various military and commercial products^24,25^. For example, they have been used in catalyst support and microelectronics^26^ and in semiconductor materials, glass products, rocket fuel, explosives, wear-resistant coatings for ships, packaging materials, cosmetic fillers, plastics and sensors^27–29^. The phytotoxicity of Al_2_O_3_ NPs was reported in tobacco and wheat^30,31^. Lee *et al*.^32^ investigated the phytotoxicity of Al_2_O_3_ NPs (∼150 nm) to *Arabidopsis thaliana* at concentrations of 400, 2,000 or 4,000 mg/L and no toxic effects were observed^32^. Some positive effects of Al_2_O_3_ NPs were reported on the root growth of ryegrass, radish, lettuce and rape plants^33^. Riahi-Madvar *et al*.^31^ showed that antioxidant enzyme activities of wheat plant decreased the damaging effects of Al_2_O_3_ NPs by scavenging the induced ROS^31^.

Nickel oxide nanoparticles (NiO NPs) have many novel properties compared to their bulk counterpart and are widely used in catalytic materials, sensors, magnetic materials, battery electrode, diesel–fuel additives and electrochromic films^34,35^. It has been reported that NiO NPs can be easily transported into plants inducing cytotoxic and genotoxic effects^36^. Feisal *et al*.^37^ reported that NiO NPs at 0.025–2.0 mg/mL led to oxidative stress, mitochondrial dysfunction, lipid peroxidation and apoptosis/necrosis in tomato seedling roots^37^. The activity of antioxidative enzymes, including CAT, and SOD was improved. Dewez *et al*.^38^ indicated that both NiO NPs and bulk NiO at 1000 mg/L increased ROS generation and had high inhibitory effect on the PSII quantum yield, reducing the photosynthetic electron transport performance in *Lemna gibba* L.^38^.

The aims of this work were to investigate the impact of Al_2_O_3_ and NiO NPs on the growth and antioxidative responses of *Nigella arvensis* at different concentrations. *N. arvensis* is a grassy plant with small black seeds known as the “seed of blessing”. The plant belongs to the genus of *Nigella* and family of Ranculaceae. The seeds have been used to enhance flavor in cakes and bread. They are used as curative material for treating various diseases in some parts of the world. The seeds also demonstrate some therapeutic (for worm infestation), antiallergic, antiviral and anti-inflammatory properties^39,40^. In this study, we investigated the roles of Al_2_O_3_ and NiO NPs on plant growth, intracellular ROS generation and levels of antioxidant responses for example activity of different antioxidant enzymes including CAT, APX, SOD and POD, as well as the alteration of secondary metabolites such as total phenolic, total iridoid and total saponin content, total antioxidant capacity, total reducing power and scavenging activity against DPPH free radical in the treated medicinal plant of *N. arvensis*.

## Results and Discussion

### Biomass assay

**Figure 1:**
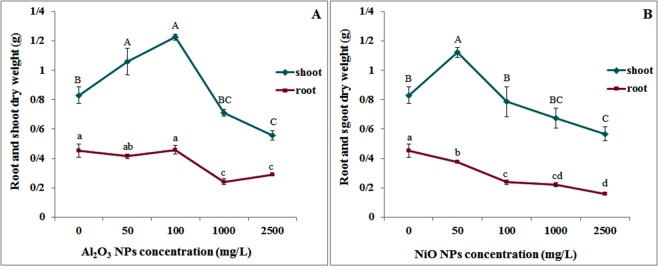
Effects of (**A**) Al_2_O_3_ NPs and (**B**) NiO NPs treatment on plant biomass of *N. arvensis*.

The results (Fig. 1A) showed that Al_2_O_3_ NPs up to 100 mg/L stimulated the growth of *N. arvensis*, but exerted toxicity at higher concentrations. The highest shoot and root dry weight (1.2 ± 0.02 and 0.46 ± 0.03 g) and the lowest shoot and root weight (0.55 ± 0.03 and 0.28 ± 0.002 g) was observed at 100 and 2500 mg/L Al_2_O_3_ NPs respectively. NiO NPs demonstrated significantly stronger effects than Al_2_O_3_ NPs on the growth of *N. arvensis*. Maximum shoot and root dry weights (1.12 ± 0.03 and 0.37 ± 0.009 g respectively) were obtained at 50 mg/L, and the lowest shoot and root dry weight (0.56 ± 0.04 and 0.15 ± 0.007 g) were found at 2500 mg/L NiO NPs (Fig. 1B). Consistent with our results, Asztemborska *et al*.^41^ also reported an increase in plant biomass of *Allium cepa L*., *Zea mays*, *Lepidium sativum* and *Kalanchoe daigremontiana* upon exposure to low concentrations of Al_2_O_3_ NPs, but a 33% decrease of root dry mass was noticed at the highest Al_2_O_3_ NPs concentration (1000 mg/kg)^41^.

### H_2_O_2_ content and antioxidant enzymes activity

**Figure 2:**
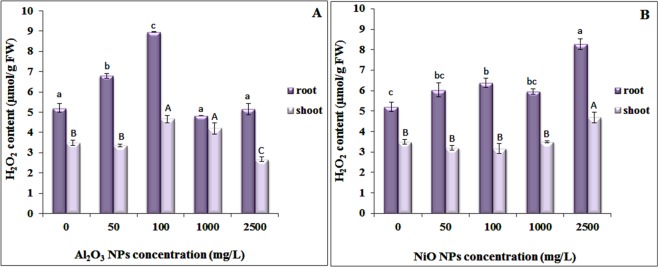
Effects of (**A**) Al_2_O_3_NPs and (**B**) NiO NPs treatment on H_2_O_2_ content in the roots and shoots of *N. arvensis*.

ROS is normally formed as a by-product of plant cellular metabolism. Various environmental stresses can lead to overproduction of ROS in plants, which can cause progressive oxidative damage. After exposure to Al_2_O_3_ NPs, H_2_O_2_ content (a product of the superoxide dismutase reaction) in the roots of *N. arvensis* increased at 50 and 100 mg/L but decreased significantly at 1000 and 2500 mg/L to a comparable level of the control plants, suggesting that excessive amounts of ROS were scavenged by antioxidant enzymes at higher concentrations. However, H_2_O_2_ content in the shoot increased with the increase of Al_2_O_3_ NPs up to 1000 mg/L, indicating higher Al stress at higher concentrations (Fig. 2A). Formation of ROS can be a result of interactions between Al and plant cells^42,43^. Pakrashi *et al*.^44^ indicated that leached out Al^3+^ and electrostatic interactions between Al_2_O_3_ NPs and algae cells might synergistically alter surface functional moieties on algal cells, resulting in oxidative stress and cell membrane damage^44^.

In *N. arvensis* treated with NiO NPs, H_2_O_2_ content in both root and shoot tissues increased significantly at 2500 mg/L compared to the control and other treatments. Exposure to 2500 mg/L NiO NPs increased the root H_2_O_2_ content by 59% compared to control, which functions as a signaling molecule in the induction and regulation of antioxidants. The H_2_O_2_ content in the shoots exposed to NiO NPs increased significantly by 34% at 2500 mg/L compared to the control, but was unaffected in other treatments (Fig. 2B). Other studies with *Vicia narbonensis* L.^45^ and *Zea mays*^19^ treated with titanium oxide (TiO_2_) and cerium oxide (CeO_2_) NPs demonstrated little effects of these NPs on H_2_O_2_ accumulation in plant tissues, indicating that the induction of H_2_O_2_ in plants vary with the properties (e.g. composition) of metallic nanoparticles.

**Figure 3:**
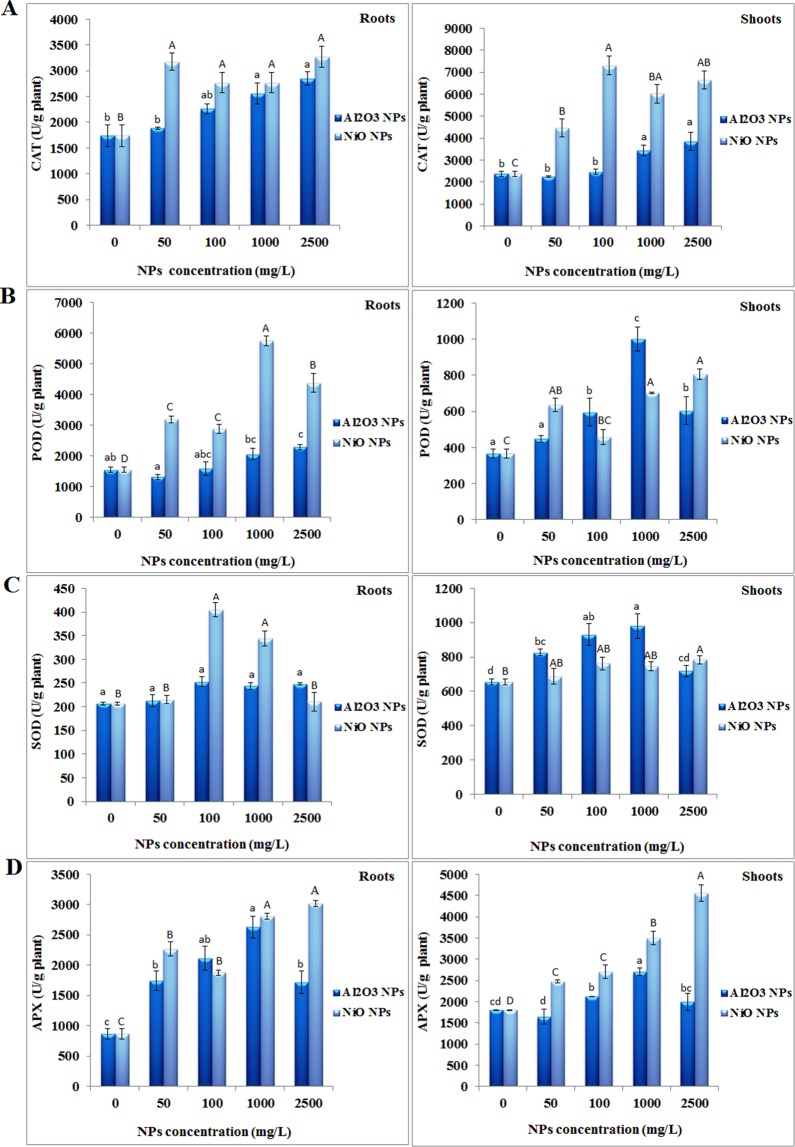
Effects of Al_2_O_3_ and NiO NPs treatment on antioxidative enzymes: (**A**) CAT, (**B**) POD, (**C**) SOD and (**D**) APX activity in the roots and shoots of *N. arvensis*.

The activities of antioxidant enzymes in plants increase under environmental stresses^46^. Enhanced activities of antioxidant enzymes can increase plant tolerance to oxidative stress^47^. Figure 3 shows the activities of antioxidant enzymes in the roots and shoots of *N. arvensis* in the presence of 0–2500 mg/L Al_2_O_3_ and NiO NPs. The CAT activity in the shoots and roots of *N. arvensis* increased with increasing ENPs levels for both nanoparticles (p < 0.01) (Fig. 3A), but the patterns of increase differed between these two ENPs. The CAT activity in plant tissues significantly increased after exposure to different concentrations of Al_2_O_3_ NPs compared to the control plants, however, the differences between treatments with different concentrations of Al_2_O_3_ NPs are generally insignificant. By contrast, the CAT activity displayed a dose-response relationship with NiO NPs, with higher NiO NPs leading to greater CAT activity in both roots and shoots (Fig. 3A). The exposure to CAT is one of the most important enzymes that scavenge ROS in plant cells. CAT partakes in the main defense system against the increase of H_2_O_2_ and can regulate the H_2_O_2_ levels in cells by converting it to water and oxygen^48^. The increased activity with increasing ENPs concentrations correlated with the decreased H_2_O_2_ content and lipid peroxidation in plants (Fig. 2), underscoring the importance of CAT in alleviating ENPs-induced oxidative stress. Furthermore, higher activity of CAT can be attributed to higher activities of SOD and higher production of H_2_O_2_. In agreement with our observations in roots and shoots, Laware and Raskar, (2014) found that the CAT activity in plants was increased upon exposure to 300 mg/L TiO_2_ NPs^49^. Also consistent with our previous results, CAT activity in tomato roots was not affected by up to 250 mg/L NiO NPs treatment, but its activity increased significantly in treatments with 250 to 2000 mg/L of NiO NPs^37^. Interestingly, TiO_2_ at 100 and 200 mg/L concentrations actually reduced the CAT activity compared with the control^49^, indicating that ENP composition plays a role in the induction of oxidative stress in plants.

The POD activities in *N. arvensis* roots and shoots were elevated after exposure to both Al_2_O_3_ and NiO NPs. The POD activity in plant roots increased by 1.32 and 1.47 fold at 1000 and 2500 mg/L of Al_2_O_3_NPs (p < 0.05) compared to the control. Similarly, NiO NPs markedly increased the POD activity in plant root, with a 3.7 fold increase at 1000 mg/L. Interestingly, 2500 mg/L of NiO NPs significantly decreased the POD activity in plant roots by 24% compared with the 1000 mg/L treatment (P < 0.05).The enhancement of POD activity in plant shoot was relatively mild compared with that in plant roots. Treatment with 1000 and 2500 mg/L NiO NPs resulted in 1.9 and 2.2 fold increase of POD activity in plant shoots (Fig. 3B). In addition to reducing H_2_O_2_ accumulation during oxidative stress, POD also affects lignin and ethylene synthesis, as well as the decomposition of indole-3-acetic acid (IAA). It also involves in plant resistance against pathogens and wound healing^48^. The increase in POD activities against studied ENPs implies the protective ability of *N. arvensis* against oxidative stress. In agreement with our studies, increased POD activity in *Glycine max* and *Cucumis sativus* treated with CuO NPs, in *Triticum aestivum* treated with Ag NPs, and in *Vicia narbonensis* treated with TiO_2_ NPs was also reported^23,45,50,51^.

SOD plays an important role against ROS-mediated toxicity by catalyzing the dismutation of free hydroxyl radicals to H_2_O_2_ and O_2_. Al_2_O_3_ and NiO NPs displayed different effects on SOD activity in the shoots and roots of *N. arvensis*. The presence of NiO NPs up to 1000 mg/L increased the SOD activity in roots, but decreased the SOD activity at 2500 mg/L to a similar level in the control plant. The SOD activities in the shoots also increased with NiO NPs, but the increase was significant only at the highest concentration of 2500 mg/L (p < 0.01). The addition of Al_2_O_3_ NPs had no significant effects on SOD activities of *N. arvensis* roots. When *N. arvensis* was treated with 1000 mg/L of Al_2_O_3_ NPs, the SOD activity in plant shoots was significantly enhanced by 49% (p < 0.05), while 2500 mg/L of this ENP reduced shoot SOD activity by 27% (p < 0.01) compared with the 1000 mg/L treatment (Fig. 3C). Significant increase in SOD activity may be due to either direct effect of these ENPs on the SOD gene expression or an indirect effect mediated through an increase in the level of O_2_^−•^ radicals. Rajeshwari *et al*.^52^ found similar impact of Al_2_O_3_ NPs on SOD activity in *Allium cepa* root tips and a maximum increase was found at 100 mg/L^52^. Feisal *et al*.^37^ revealed that in NiO NPs treated tomato roots, SOD activity increased with increasing NiO NPs in comparison to control but its activity was decreased at 1500 and 2000 mg/L NiO NPs that confirms our results^37^. Therefore, reduced SOD activity in this study under the highest Al_2_O_3_ and NiO NPs concentrations may reflect the low ROS scavenging capacity and increased damage to plants^11^.

As a member of the ascorbic acid-glutathione cycle, APX plays a crucial role in eliminating hazardous H_2_O_2_ from plant cells. There was a progressive increase in APX activity with increasing NiO NPs in the shoots and roots of *N. arvensis*. The APX slightly increased with the increase of NiO NPs from 50–100 mg/L, but substantially increased in 1000 and 2500 mg/L NiO NPs treated plants. In plants treated with 2500 mg/L of NiO NPs, APX activity in the roots and shoots of *N. arvensis* was 3.49 and 2.54-fold of their respective controls (0 mg/L) (Fig. 3D). The APX activity in plants exposed to Al_2_O_3_ NPs exhibited different patterns in comparison to its activity under NiO NPs exposure. The enzyme activities were increased gradually with increasing level of Al_2_O_3_ NPs, and reached the maximum at 1000 mg/L. The APX activity decreased at the Al_2_O_3_ NPs concentration of 2500 mg/L (Fig. 3D).

By comparing the activities of these four enzymatic antioxidants (CAT, POD, SOD and APX), it is evident that the accumulation of studied ENPs induced a strong antioxidant response in *N. arvensis*. Furthermore, these results showed differential responses of the antioxidant enzymes to different ENPs in different plant tissues, probably stemming from the different physico-chemical properties of these two ENPs such as their size, shape, surface chemistry.

### Antioxidant compounds

**Figure 4:**
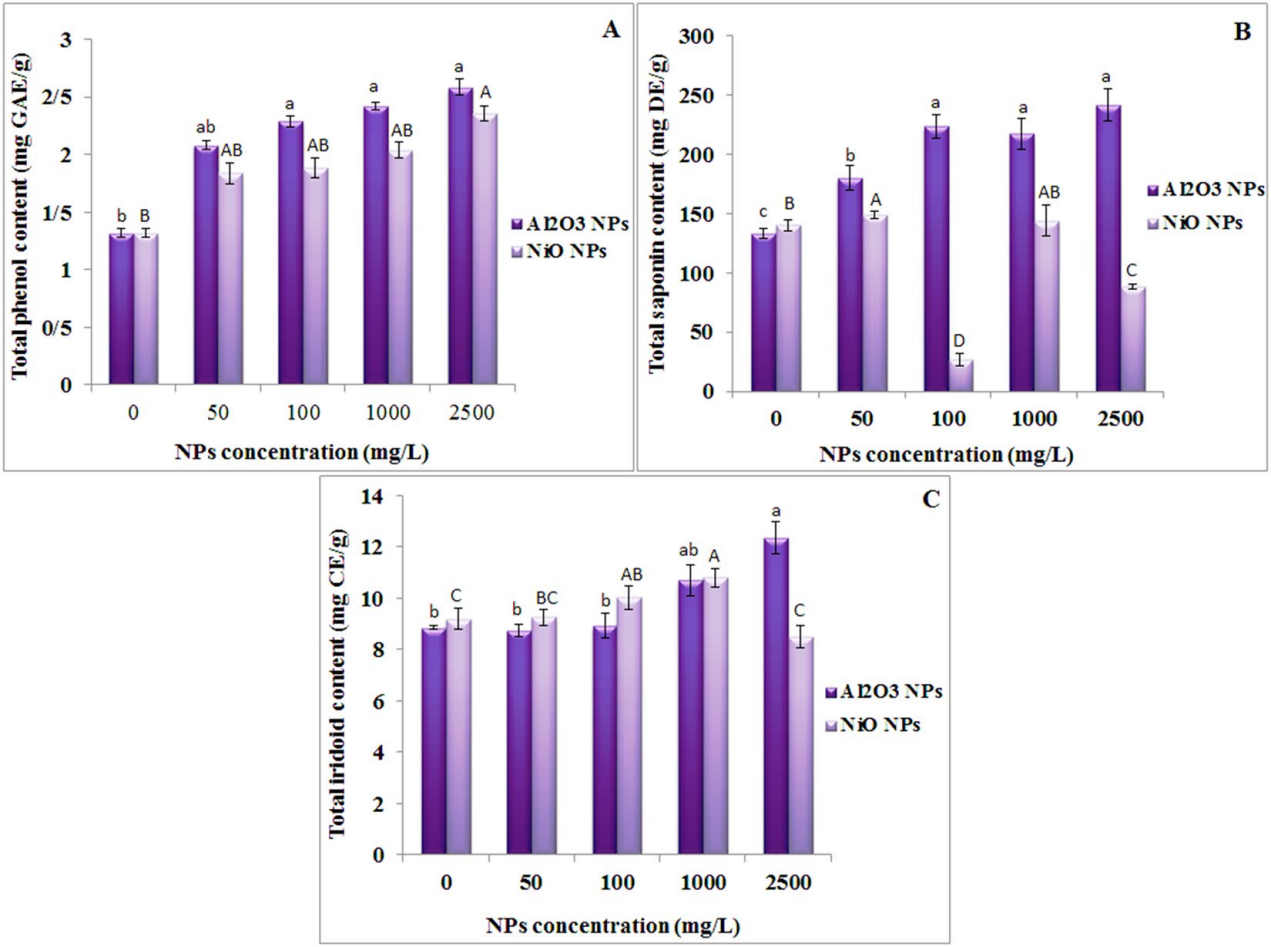
Effects of Al_2_O_3_ and NiO NPs treatment on secondary metabolites activity: (**A**) total phenols, (**B**) total saponins and (**C**) total iridoids in shoots of *N. arvensis*.

Secondary metabolites such as the total phenols, saponins and iridoids were measured in the shoots of *N. arvensis*. Phenols as secondary metabolites of plants are known to be involved in the antioxidant activity in plants growing under heavy metal stress and are typically increased by metal stress^53^. Also, phenols are oxidized by peroxidase and play a role in scavenging H_2_O_2_ molecules^54^. In this study, both ENPs enhanced the total phenol contents in *N. arvesis* shoot compared with the control and there was no significant difference between the treatment groups of between 50–2500 mg/L NiO NPs. As shown in Fig. 4A, the total phenol content was significantly increased by 96% and 79% in the presence of 2500 mg/L of Al_2_O_3_ and NiO NPs respectively in comparison to the control plants. The effects were more pronounced in the shoots of Al_2_O_3_ NPs treated plants than NiO NPs treated plants (Fig. 4A). Similar effects were also recorded in *Eichhornia crassipes*^55^ and *Bacopa monnieri*^56^ under Ag NPs, which supports the conviction that ENPs induce the production of total phenolic compounds in plants.

Saponin is a class of amphipathic glycosides, which have one or more hydrophilic glycoside moieties combined with a lipophilic triterpene derivative^57^. The total saponin content of plants treated with Al_2_O_3_ and NiO NPs at various concentrations is shown in Fig. 4B. Al_2_O_3_ NPs significantly increased the total saponin production in *N. arvensis* at all tested concentrations, whereas its content dropped 5 and 1.6 times at 100 and 2500 mg/L NiO NPs, compared with the control. This result agrees with a previous report that low concentrations of Ag NPs increased total saponin content in *Calendula officinalis*^58^, but higher Ag NPs (0.8 to 1.6 mM) decreased the total saponin content in the same plant.

Iridoids are a type of monoterpenes, which are typically found in plants as glycosides, often bound to glucose. The iridoids produced by plants act primarily as a defense against biotic and abiotic stresses. A marked increase (39% compared with controls, Fig. 4C) in total iridoids concentration was observed in plant shoots treated with 2500 mg/L Al_2_O_3_ NPs. However, shoot iridoids content steadily increased in the presence of up to 1000 mg/L NiO NPs before decreased in plants treated with 2500 mg/L NiO NPs.

### Total antioxidant capacity, DPPH scavenging and reducing power activities

**Figure 5:**
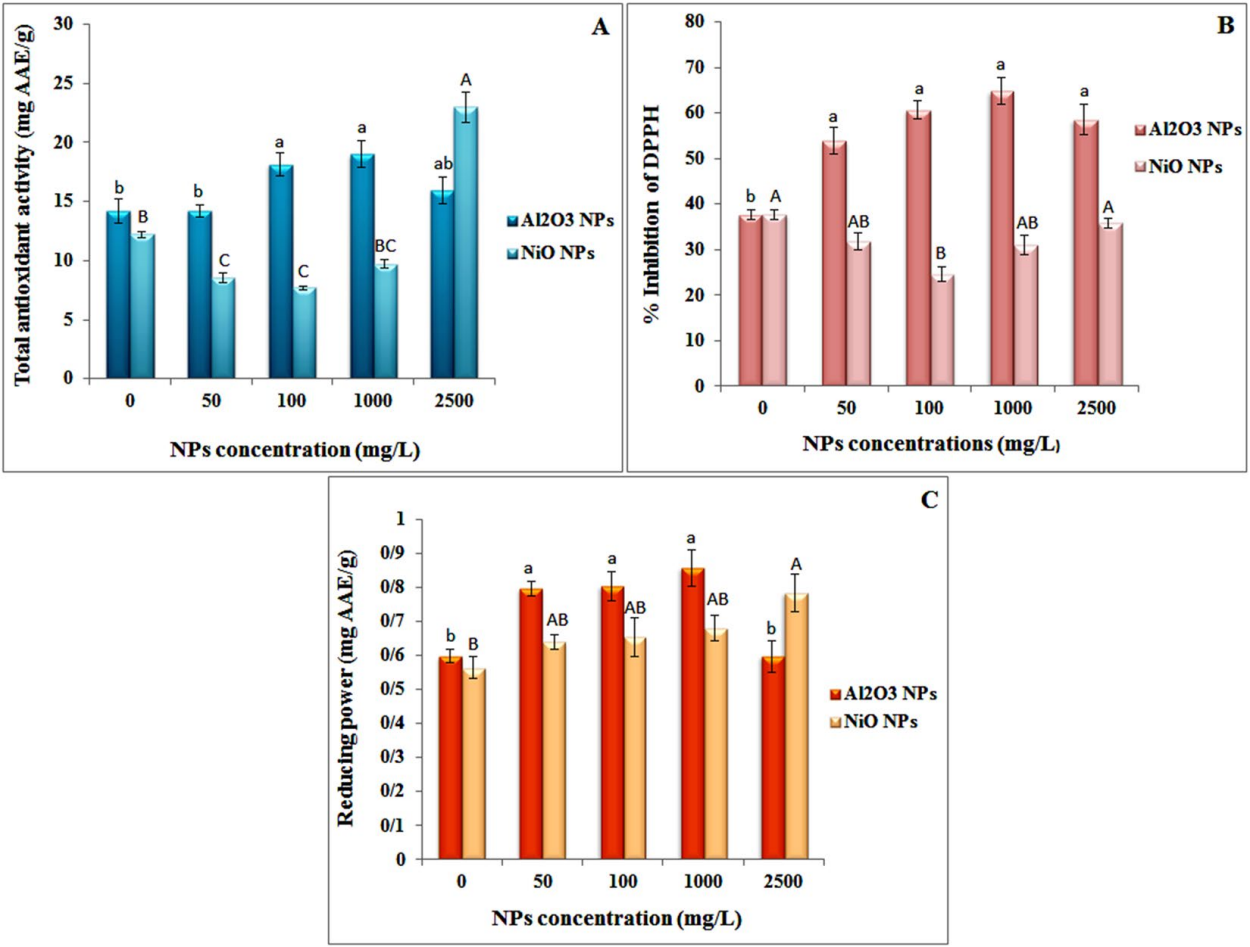
Effects of Al_2_O_3_ and NiO NPs treatments on antioxidant capacity: (**A**) total antioxidant, (**B**) DPPH scavenging, (**C**) reducing power, in shoots of *N. arvensis*.

The total antioxidant capacity of *N. arvensis* exposed to both ENPs is shown in Fig. 5A. The total antioxidant activity increased significantly at 100 and 1000 mg/L Al_2_O_3_ NPs. Plant exposure to 0–1000 mg/L of NiO NPs resulted in a decrease of the total antioxidant capacity compared with plants grown in ENPs-free medium (Fig. 5A). By contrast, the total antioxidant capacity was significantly increased at 2500 mg/L NiO NPs (88.5% increase over control).

In this study, DPPH free radical scavenging activity in shoot of *N. arvensis* exposed to NiO NPs increased with increasing NiO NPs concentrations after three weeks of treatment (Fig. 5B). When *N. arvensis* was exposed to 200 µL of 2500 mg/L of NiO NPs, the DPPH free radical scavenging activity reached the maximum of 35.79% of inhibition (Fig. 5B). This result implies that *N. arvensis* has a high capability to cope with the oxidative stress induced by NiO NPs. In plants exposed to Al_2_O_3_ NPs, DPPH scavenging activity increased in different levels with increasing Al_2_O_3_ NPs concentrations from 50 to 1000 mg/L (Fig. 5B). The highest DPPH free radical scavenging activity in plant shoot was 4.93 fold of the control at 1000 mg/L Al_2_O_3_ NPs. The DPPH free radical scavenging activity were decreased by 17% in extracts containing 2500 mg/L of Al_2_O_3_ NPs compared with 1000 mg/L treated plants. Similar to our results, Khan *et al*.^59^ evaluated the effects of nine types of metal ENPs including monometallic and bimetallic alloy ENPs such as AgCu, AuCu and AgAu on DPPH free radical scavenging activity of *Silybum marianum*^58^. They showed an increase in DPPH percentage after 28 days of exposure for all ENPs suspensions. Javed *et al*.^60^ demonstrated that DPPH free radical scavenging activity in *Stevia rebaudiana* Bertoni shoots was 74.8% and 68.6% higher under 10 mg/L and 1 mg/L ZnO NPs treatments^60^. Their results also showed that the lowest antioxidant activities were obtained from extracts containing 1000 mg/L ZnO NPs in MS medium, which can be related to the toxic effects of this treatment by generating oxidative stress and imbalance of anti-oxidative activities^60^.

The reduction of Fe (III) is often used as an indicator of electron-donating activity, an important process in phenolic antioxidant reaction^61^. The presence of reductant (antioxidant) in the plant extracts causes the reduction of the Fe^3+^/ferricyanide complex to the ferrous form. Therefore, the concentration of Fe^2+^ was monitored by measuring the formation of Perl’s Prussian blue at 700 nm^62^. In this study, the total reducing power of plants exposed to both Al_2_O_3_ and NiO NPs was affected similarly as the DPPH free radical scavenging activity (Fig. 5C). Although these effects were mostly not statistically significant (p > 0.05), there were significant (p < 0.05) increases in the total reducing power of shoot extract treated with 1000 mg/L of Al_2_O_3_ NPs (by 43%) and those treated with 2500 mg/L of NiO NPs (by 32%) when compared to the control plants. Therefore, with Al_2_O_3_ NPs treatments, the lowest antioxidant activities were obtained from extracts containing 2500 mg/L NPs treatments, which also had lower DPPH free radical scavenging activity and total reducing power activity than the control group.

## Conclusion

In summary, this study demonstrated the concentration dependent effects of NiO and Al_2_O_3_ NPs on the growth and antioxidant activities of *N. arvensis*. NiO NPs exhibited greater effects than Al_2_O_3_ NPs on *N. arvensis* growth. Significantly enhanced activities of antioxidant enzymes (CAT, POD, APX and SOD) and antioxidant compounds (total iridoids, total saponin, and total phenolic) along with DPPH scavenging activity, total antioxidant capacity and total reducing power were observed in plants treated with 50 to 1000 mg/L of NiO and Al_2_O_3_ NPs in hydroponic systems. However, adverse effects of NiO and Al_2_O_3_ NPs on these phytochemical assays appeared when Hoagland medium was supplemented with 1000 or 2500 mg/L of NiO and Al_2_O_3_ NPs. The concentration and composition-dependent responses of plants observed in this study provide new insights into the effects of ENPs in the mineral nutrition, antioxidant activities and alteration of metabolic pathways of medicinal plants.

## Materials and Methods

### Nanomaterials, chemicals and seeds

Al_2_O_3_ and NiO NPs were purchased from Iranian Nanomaterial Company. According to the supplier, the diameter of NiO NPs fell in the range 5–8 nm. Most NiO NPs were spherical and has the purity of 99.5%. The specific surface area was in the range of 50–100 m^2^/g. The average diameter of Al_2_O_3_ NPs was about 5 nm. The purity of Al_2_O_3_ NPs was 99.99% and its specific surface area was 150 m^2^/g. Seeds of *N. arvensis* were purchased from Pakanbazr Company (Isfahan, Iran).

### Preparation of particles and cultures

Seeds of *N. arvensis* were sterilized for 10 min in 10% sodium hypochlorite solution before germination. They were germinated on sand soaked with 0.1 strength modified Hoagland solution (0.5 mM MgSO_4_, 2.5 mM Ca (NO_3_)_2_, 0.5 mM KH_2_PO_4_, 2.5 mM KCl, 25 μM H_3_BO_3_, 5 μM MnSO_4_, 0.4 μM ZnSO_4_, 0.2 μM CuSO_4_, 0.25 μM Na_2_MoO_4_, 50 μM Fe-EDTA, pH 5.5). After 10 days, the solution was replaced with Hoagland solutions containing different concentrations of Al_2_O_3_ or NiO NPs (0, 50, 100, 1000 and 2500 mg/L). Prior to the replacement, the ENP suspensions (100 mL) were first sonicated in an ultrasonic water-bath for 90 min. All experiments were carried out in a greenhouse under semi-controlled conditions: day/night photoperiod (16/8 h), a light intensity of 100 μM/m²/s, day/night temperature (24/20 ± 1 °C) and day/night relative humidity (70/75%). Hydroponic system was adopted for this study to avoid the compounding effect of soil particles and microorganisms in soil on the physiological effect of chosen nanoparticles. In addition, hydroponic system is gain popularity in urban agriculture for vegetable growth due to dwindling global arable lands. Therefore, the results hold great importance for sustainable applications of nanotechnology in agriculture. A wide range of concentrations are used for both ENPs so that the physiological responses of plants in the presence of mild to severe contamination can be investigated. All treatments had three replicates and the experiment lasted for three weeks. Afterwards, the plants were harvested, and rinsed with tap water. The shoot and root biomass were measured after they were oven dried at 65 °C for three days.

#### Hydrogen peroxide (H_2_O_2_) content

Hydrogen peroxide level was measured following the method described by Sergiev *et al*.^63^. Fresh roots or shoots (0.5 g) were homogenized in ice bath with 5 mL of 0.1% (W/V) trichloroacetic acid (TCA). The mixtures were centrifuged at 12,000 g for 15 min. 0.5 mL of the obtained supernatant was added to the mixture containing 0.5 mL of potassium phosphate buffer (10 mM, pH 7.0) and 1 mL of KI solution (1 M). The content of H_2_O_2_ was determined with a spectrophotometer (Bausch & Lomb 70) at 390 nm.

#### Enzyme extractions and assays

To prepare extracts for the analysis of enzyme activities, fresh plant tissues were ground to fine powders in liquid nitrogen and extracted at a ratio 1:3 (w/v) fresh weight to extraction buffer containing 1 mM EDTA, 3 mM DTT and 5% PVP. The homogenate thus obtained was centrifuged for 20 min at 14,000 rpm and the supernatant was either used for enzyme assays or protein extraction, which is described below in details.

#### Catalase (CAT) activity

Catalase activity was determined according to Aebi’s (1984) method^64^. 0.1 mL of enzyme extract was mixed with 2.9 mL of 50 mM phosphate buffer (pH 7.0) containing 30 mM H_2_O_2_ to make a total volume of 3 mL. CAT activity was estimated based on the decreased absorbance of H_2_O_2_ at 240 nm (Shimadzu-UV mini- 1240). The CAT activity was determined based on the molar extinction coefficient of H_2_O_2_ (39.4 M^−1^cm^−1^) and is expressed as µM H_2_O_2_ per mg fresh weight per min.

#### Ascorbate peroxidase (APX) activity

Ascorbate peroxidase was assayed based on the method reported by Nakano and Asada (1981)^65^. The reaction complex contained potassium phosphate (50 mM, pH 7.0), EDTA (0.2 mM), ascorbic acid (0.5 mM), 2% H_2_O_2_, and 100 µL of enzyme extract with a total volume of 3 mL. A reduction of absorbance (at 290 nm for 1 min) was recorded. The activity of enzyme was estimated using the extinction coefficient of 2.8 mM^−1^ cm^−1^. One unit APX activity was defined as 1 mM ascorbate oxidized per mL per min at 25 °C.

#### Peroxidase (POD) activity

Peroxidase activity was measured using the guaicol oxidation method of Chance and Machly (1955)^66^. The reaction mixture (3 mL in final volume) contains potassium phosphate buffer (10 mM, pH 7.0), guaicol (8 mM) and 100 µL enzyme extract. The reaction was initiated by adding 10 µL of 40 mM H_2_O_2_. The absorbance was determined spectrophotometerically (Bausch & Lomb 70) within 1 min at 470 nm. POD activity was calculated using the extinction coefficient of 26.6 mM^−1^ cm^−1^.

### Superoxide Dismutase (SOD) activity

Superoxide dismutase activity was determined following the Giannopolitis and Ries (1977) protocol by spectrophotometer method^67^. In this method, reaction solution contained 13 mM methionine, 75 μM nitroblue tetrazolium (NBT), 2 μM riboflavin, 50 mM phosphate buffer (pH = 7.8) and 0–50 μL of extracted enzyme. Reaction was started by placing the tubes against two fluorescent lamps (15 W). The reaction was terminated after 10 min by turning off the lamps. Absorption was measured at 560 nm with a spectrophotometer (Bausch & Lomb 70). The illuminated and non-illuminated reactions without supernatant were placed as calibration standards. Ultimately, one unit of enzyme activity was equivalent to the amount of the enzyme needed for 50% reduction of the NBT photochemical reaction.

### Preparation of extract and antioxidant assays

*N. arvensis* leaf extracts were prepared by drying and then grounding leaves from plants exposed to various concentrations of ENPs. One gram of leaf powder from each treatment was ground and extracted by 3 ml of acidic methanol reagent (99:1 methanol: HCl) in 25 ^°^C for 24 h and the extract was centrifuged at 10,000 rpm for 15 min. The supernatant was collected and then stored in an airtight container in the refrigerator (4 °C) for the measurement of all antioxidant compounds. The spectrophotometric assays were performed in triplicates.

### Determination of total phenolic content

The total phenols were determined based on the Folin–Ciocalteu method^68^. An aliquot (0.5 mL) of plant extract (1 mg/mL) was mixed with 2.5 mL of Folin–Ciocalteu reagent (diluted 1:10) and 2 mL of NaHCO_3_ (7.5%). Then, the test solution was maintained at 45 °C for 15 min. The absorbance was recorded at 765 nm with a spectrophotometer (Bausch & Lomb 70) against a blank sample. Total phenols were determined as Gallic acid (standard) equivalents (mg GA/g extract).

### Total saponins assay

Total saponin contents in leaves were estimated using the colorimetric method reported by Hiai *et al*.^69^ with minor modifications^69^. 50 µL of plant extract was added to different test tubes containing 0.25 mL of vanillin reagent (8% w/v in ethanol 99.9%). Those test tubes were placed in ice-cold water bath and 2.5 mL of 72% (v/v) sulfuric acid was slowly added to each tube. After 3 min, the tubes were heated to 60 °C for 10 min using a water bath and then cooled to room temperature. Absorbance was measured at 544 nm using a spectrophotometer against the reagent blank. Diosgenin was used as a standard and the content of total saponins was expressed as Diosgenin equivalents (mg DE/g extract).

### Estimation of total iridoid content

The total iridoid content was determined according to the colorimetric method described by Haag-Berrurier *et al*.^70^ with slight modifications^70^. A 100 µL aliquot of plant extract solution was incubated with 900 µL of reagent solution containing 82 mL methanol, 100 mg vanillin and 8 mL concentrated sulfuric acid. Absorbance was read spectrophotometrically at 538 nm. Catalpol was used as a standard compound for the establishment of the calibration curve. Total iridoids was expressed as Catalpol equivalents (mg CE/g extract).

### Total antioxidant capacity

The capacity of total antioxidant of the methanolic extracts of samples was determined using a previously reported method by Prieto *et al*.^71^ with slight modifications^71^. An aliquot of 0.3 mL of sample extracts solution was mixed with 2.7 mL of reagent solution containing sulfuric acid (0.6 M), sodium phosphate (28 mM) and ammonium molybdate (4 mM). The tubes containing reaction solution were incubated at 95 °C for 90 min and then cooled to room temperature. All samples were run in triplicates and their absorbance was read at 695 nm by a spectrophotometer. The standard reference was ascorbic acid and the total antioxidant capacity was expressed as mg of ascorbic acid equivalent per gram of the dry extract.

### DPPH radical scavenging activity analyze

The free radical scavenging activity of plants was calculated using the method described by Sarker^72^. Briefly, serial dilutions were carried out with the stock solution (1 mg/mL) of the extracts. Diluted solutions (1 mL of each samples) were reacted with 1 mL of a freshly prepared DPPH (2,2-diphenyl-1-picryl hydrazyl) methanol solution (80 µg/mL) for 30 min in the dark at room temperature. Absorbance values of these solutions were determined with a spectrophotometer at 517 nm. Methanol was used as a blank. Control sample was prepared containing the same amount of methanol and DPPH without test compounds. Inhibitions of DPPH radical in percent (I%) were estimated as follow:

$${{\rm{I}} \% =[({\rm{A}}}_{{\rm{Control}}}\,-\,{{\rm{A}}}_{{\rm{sample}}}{)/{\rm{A}}}_{{\rm{Control}}}]\times 100$$

where A_Control_ is defined as the absorbance of the control reaction (comprising all reagents without the test compound) and A_sample_ is the absorbance of the test compounds.

### Reducing power activity

Total reducing power activity of samples was investigated according to the method described by Aman *et al*.^73^ with slight modifications^73^. An aliquot of 2.5 mL of stock solution of each sample was mixed with phosphate buffer (2.5 mL, 0.2 M, PH 6.6) and potassium ferricyanide (2.5 mL, 1%). The tubes containing the reaction solutions were then incubated at 50 °C for 20 min. Afterwards, 2.5 mL of 10% trichloroacetic acid was added to each tube and then 2.5 mL of the reaction mixture was mixed with distilled water (2.5 mL) and ferric chloride (0.5 mL, 0.1%). The absorbance of samples was measured at 700 nm with a spectrophotometer. Ascorbic acid was used as a positive control and the results were expressed as mg ascorbic acid equivalent per gram.

### Statistical analysis

The data represent mean of three replicates ± standard error (S.E). One-way ANOVA was employed to confirm the variability of data and validity of results with different rates of NPs addition. Duncan’s multiple range test (DMRT) was employed to determine the significant differences between treatments to a significance level of P < 0.05 or very significant as P ≤ 0.001. Statistical analyses were performed using SPSS (24) software.

## Data Availability

All data generated or analyzed during this study are included in this published article (and its Supplementary Information files).
